# Hypertension contributes to exacerbated osteoarthritis pathophysiology in rats in a sex-dependent manner

**DOI:** 10.1186/s13075-022-02966-9

**Published:** 2023-01-12

**Authors:** Taylor D. Yeater, Jacob L. Griffith, Carlos J. Cruz, Folly M. Patterson, Jessica L. Aldrich, Kyle D. Allen

**Affiliations:** 1 J. Crayton Pruitt Family Department of Biomedical Engineering, University of Florida, 1275 Center Drive, Biomedical Sciences Building, Gainesville, FL 32610 USA; 2 Pain Research & Intervention Center of Excellence, University of Florida, Gainesville, FL USA; 3 Department of Community Dentistry & Behavioral Sciences, University of Florida, Gainesville, FL USA; 4 Department of Orthopedics and Sports Medicine, College of Medicine, University of Florida, Gainesville, FL USA

**Keywords:** Osteoarthritis, Hypertension, Autonomic nervous system

## Abstract

**Background:**

Hypertension is a common comorbidity of osteoarthritis (OA) with known autonomic dysregulation; thus, the autonomic nervous system may provide a shared underlying mechanism. The objective of this study was to examine the role of the autonomic nervous system in a preclinical model of OA and hypertension.

**Methods:**

Experiments were conducted in spontaneously hypertensive rats and a normotensive control strain, including male and female rats. OA was surgically induced via medial meniscus transection with skin incision used as a sham control (*n* = 7–8/strain/sex/surgery). Tactile sensitivity, anxiety-related behavior, and serum corticosterone were measured at baseline then bi-weekly across 8 weeks. At weeks 9–10, cardiovascular responses to a chemical vagal nerve agonist were determined to indirectly evaluate vagus nerve function. The joint structure was assessed via grading of histological sections.

**Results:**

In males, OA resulted in thinner cartilage in both hypertensive (OA vs. non-OA *p* < 0.001) and normotensive (OA vs. non-OA *p* < 0.001). Only females with comorbid hypertension and OA displayed thinner cartilage (*p* = 0.013). Male hypertensive OA animals had increased calcified subchondral bone compared to normotensive OA animals (*p* = 0.043) while female hypertensive OA animals had increased calcified subchondral bone compared to hypertensive sham animals (*p* < 0.001). All MCLT+MMT groups developed low-grade synovitis; interestingly, hypertensive OA females had higher synovitis scores than normotensive OA females (*p* = 0.046). Additionally, hypertension led to larger drops in blood pressure with vagal activation in both OA (hypertensive vs. normotensive *p* = 0.018) and sham (hypertensive vs. normotensive *p* < 0.001) male animals. In females, this trend held true only in OA animals (normotensive vs. hypertensive *p* = 0.005).

**Conclusion:**

These data provide preliminary evidence that hypertension influences OA progression and encourages further study into the autonomic nervous system as a possible mechanism.

**Supplementary Information:**

The online version contains supplementary material available at 10.1186/s13075-022-02966-9.

## Background

The hallmark of osteoarthritis (OA) pathophysiology is a maladaptive joint repair that leads to structural changes in joint tissues, such as cartilage degradation, bone marrow sclerosis, and osteophyte formation [1]. Moreover, pain is the prevailing symptom; however, radiographic evidence of joint damage is a poor predictor of symptoms [2]. The discordance between radiographic evidence of OA and joint pain may arise from an incomplete understanding of the complex interactions between multiple organ systems beyond the joint. The OA research community has recently pushed to consider OA as a “whole-body” disease to better understand these interactions [3]. Uncovering new relationships that explain interactions between organ systems may lead to novel disease-modifying OA drugs, unveil early diagnostic markers of OA, or provide new explanations for comorbid presentations of diseases, including shared risk factors.

Toward this goal, autonomic nervous system (ANS) dysregulation has been hypothesized to contribute to OA progression through both neuronal and non-neuronal mechanisms [4–7]. The ANS is responsible for involuntary actions throughout the body and is composed of the sympathetic and parasympathetic branches. Importantly, the ANS acts as a regulator of the immune system, vascular tone, and cardiac function. The sympathetic branch acts primarily in stressed and aroused states, with the parasympathetic branch working reciprocally to balance body functions. Furthermore, the ANS interacts with the hypothalamic pituitary adrenal (HPA) axis to regulate inflammation throughout the body via cortisol release [8]. Disruptions to ANS balance have been implicated in multiple disease states, including chronic pain [9–11], rheumatoid arthritis [12, 13], and hypertension [14]. Prior work from our group has demonstrated cardiovascular changes in a rat surgical model of OA [15]. Here, joint injury in male Lewis rats caused greater reductions in heart rate with pharmacological activation of the vagus nerve—the major parasympathetic nerve of the body. However, little is known about the influence of ANS dysregulation on OA progression and vice versa.

Studying OA progression as a “whole-body” disease and through the lens of ANS dysregulation provides unique insight into the shared mechanisms of diseases between OA and common comorbidities, such as hypertension [16]. In fact, hypertension is more prevalent in OA populations compared to matched controls (75% vs. 38%) [17]. Currently, the prevailing thought for the association between OA and hypertension is centered on shared risk factors such as age, obesity, and inactivity. However, hypertension is associated with knee OA regardless of obesity [18]. Moreover, hypertension has known bi-directional associations with autonomic dysfunction. Therefore, new understandings of the shared pathophysiology of OA and hypertension could lead to new treatments for patients with these comorbidities.

As such, the goal of this study was to evaluate OA progression in a hypertensive rat strain relative to a normotensive control. Here, we evaluated the development of OA-related joint remodeling and symptoms following surgical induction of knee OA in hypertensive and normotensive rats. Moreover, to investigate the role of the ANS in hypertension that is comorbid to OA, we assessed the altered cardiovascular responses to pharmacological stimulation of the vagus nerve. Combined, the overarching goal of these studies was to explore the role of the ANS in the comorbid development of OA and hypertension.

## Methods

### Study design

In both the spontaneously hypertensive rat (SHR) strain and the normotensive control rat strain (Sprague-Dawley), OA was induced via medial collateral ligament transection plus medial meniscus transection (MCLT+MMT), with skin incision serving as a sham control. Male and female animals were used in both strains. Behavioral measures of tactile sensitivity and anxiety, in addition to blood serum corticosterone, were collected at baseline, then bi-weekly across 8 weeks post-surgery. Surgical group assignment was conducted using an online random number generator, and all data were collected via experimenters blinded to the group. To space out endpoint evaluations, animals were equally split between four separate cohorts (*n* = 2 per strain/group/sex, *N* = 16 per cohort), with cohorts separated by 1 week. With a repeated measure design, these numbers allow for a detectable effect size (*f*) of 17% at a power of 0.8. Finally, four animals were removed from the study due to post-surgical complications, leaving *n* = 7–8 per strain/group/sex (total *N* = 60). This study design is represented visually in Supplemental Fig. 1.

### Animal acquisition and housing

Sprague-Dawley and SHR animals were obtained from Charles River (Wilmington, MA, USA) and acclimated to the University of Florida (UF) animal care facilities for at least 5 days. Animals were approximately 10 weeks old upon arrival at UF, with approximate baseline weights as follows: male Sprague-Dawley (500 g), female Sprague-Dawley (250 g), male SHR (300 g), and female SHR (150 g). At the time of surgery, animals were 12–18 weeks old. Animals were housed in pairs with their like sex and strain in a lighting-controlled environment (12-h light/dark cycle) with access to food and water ad libitum. Standard bedding and housing were used throughout the study, and all procedures were approved by the UF Institutional Animal Care and Use Committee.

### Knee surgeries

The right knees were aseptically prepared by shaving the area and cleaning with three sequential washes of betadine and 70% ethanol, ending with the fourth application of betadine. For all surgeries, an incision was made over the stifle joint, slightly medial to the midline, and the muscle was bluntly dissected to visualize the medial collateral ligament. At this point, the surgeon was informed of the surgical group. If skin incision sham, the muscle and skin were re-approximated using sterile absorbable 4-0 suture. If MCLT+MMT, the medial collateral ligament was first transected to visualize the meniscus, then the meniscus was cut in its central portion. Muscle and skin closure then occurred as described above. Due to changes in UF veterinary care recommendations during the study, cohorts received different post-operative analgesia. Cohort 1 received 4 doses of subcutaneous standard buprenorphine (1 mg/kg) delivered every 12 h; Cohort two received 6 doses of subcutaneous standard buprenorphine (1 mg/kg) delivered every 12 h plus 3 doses of meloxicam (5mg/kg) delivered daily. Cohorts 3 and 4 received 3 doses of meloxicam (5mg/kg) delivered daily and a single injection of sustained-release buprenorphine (1 mg/kg) delivered peri-operatively. While meloxicam is an anti-inflammatory drug and inflammation plays an important role in OA development, the addition of meloxicam to cohorts 2–4 was conducted in consultation with veterinary care staff and ethics board. This addition was to provide better pain control after surgery, thereby complying with the refinement principle for human-animal experimentation. As discussed in the results, there were no statistical differences in histological scoring between animals that received post-operative meloxicam and those that did not.

### Behavioral measures

All behavioral measures were collected at baseline and bi-weekly across 8 weeks post-surgery. Before baseline measurements, animals were acclimated to behavioral testing environments twice for 30 min each. Tactile sensitivity was measured on the ipsilateral hind paw using Chaplan’s up-down method for von Frey filaments [19]. For activity monitoring, animals were video recorded for 30 min while freely exploring an open field arena (35″ × 35″ × 16″). The location of the animal’s centroid was determined using the regionprops functions in MATLAB (The Mathworks, Natick, MA) on a frame-by-frame basis, thereby determining the time spent in the corners (greater anxiety behavior) and the total distance traveled by each animal during the test. Behavioral measures were conducted on different days to minimize confounding influence of the two tests on one another.

### Blood serum corticosterone

Blood serum was collected via tail vein blood draw under isoflurane anesthesia at baseline and every 2 weeks out to 8 weeks post-surgery. Blood collections were performed between 9 am and 12 pm at each time point to control for the confounding influence of circadian rhythm. Additionally, blood collections occurred on a different day from behavioral measures. Corticosterone levels were determined with an ELISA (Enzo Life Sciences, Farmingdale, NY).

### Histological evaluation of knee pathology

Operated knees were collected, fixed in 10% neutral buffered formalin for 48 h, and then stored in 70% ethanol before proceeding to decalcification. Then, the knees were decalcified in 10% formic acid for 2 weeks and infiltrated with paraffin wax via a Leica Tissue Processor at the UF Molecular Pathology Core Facility. After embedding in paraffin wax, the joints were sectioned in the frontal plane (10 μm), with sections representing the loading region of the joint being stained with safranin-o and fast green or hematoxylin and eosin. Using an open-source program developed in-house [20], user-defined regions of interest were used to calculate the minimum cartilage thickness, osteophyte area, and maximum subchondral trabecular bone area to total subchondral area (bone + marrow) in the tibial region of the medial joint compartment.

In addition to these quantitative methods, semi-quantitative scores were assigned to each joint. These scores included OARSI grades [21] or both medial and lateral compartments of the joint and synovitis score of the medial compartment [22]. Grades were assigned based on the consensus of four scorers blinded with respect to the group.

### Endpoint measures of cardiovascular function

Endpoint measures of cardiovascular function were assessed as previously described [15]. Rats were anesthetized with isoflurane, and body temperature was maintained at 37 °C. The femoral artery and vein contralateral to injury were catheterized for blood pressure measurements and drug administration, respectively. Electrocardiogram and blood pressure were recorded continuously using Grass Technologies P511 AC Amplifier and a CED Power1401 mk II data acquisition interface. Signals were collected using the CED Spike II (version 8) software. To assess cardiac autonomic function, 1-phenylbiguanide, a 5-HT3A receptor agonist with reported vagal activation properties, was administered at 100 μg/kg [23]. To confirm the action of phenybiguanide, parasympathetic influence on the heart was chemically blocked with atropine, and phenylbiguanide was again administered; 10 min was allowed between drug doses. Heart rate and blood pressure responses were analyzed as a change from the pre-stimulation value immediately preceding the injection of phenylbiguanide. Data were binned every 5 s and reported out to 30 s post-injection.

### Statistical analysis

The effect of the animal sex on cardiovascular function in the context of OA and knee surgery was unknown. As such, our study was not designed to assess the effect of animal sex; instead, separate statistical models were conducted for male and female animals. These data do, however, serve as an estimate of this interaction for future studies.

For longitudinal behavioral and corticosterone measurements, a linear mixed effects model was conducted using group, week, and the interaction of group and week as fixed effects and animal identifier as a random effect (R Studio, lme4 package). Here, the group represented a combination of surgery type and strain, as in normotensive-sham, normotensive-MCLT+MMT, hypertensive-sham, and hypertensive-MCLT+MMT; this was done to reduce the complexity of the interaction terms associated with changes across time (week). To account for any pre-surgical variation, baseline data were included as a covariate in the model. If indicated, least squared means for group, week, or the interaction term were compared using multiple comparison that was corrected for compounding type I errors using Tukey’s HSD (R Studio, lsmeans package). Longitudinal weight measurements were also compared via a linear mixed effects model that was conducted using group, week, and the interaction of group and week as fixed effects and animal identifier as a random effect. Here, two values in the female Sprague animals were misrecorded and thus not included in the analysis.

For histological grading, quantitative data were analyzed with one-way ANOVA and Tukey’s HSD post hoc tests, if the group’s main effect was significant. Semi-quantitative scores were evaluated via the Kruskal-Wallis test and Mann-Whitney *U* post hoc test, if the main effect was significant.

The endpoint pre-stimulation heart rate and blood pressure during endpoint surgeries were analyzed with one-way ANOVA and Tukey’s HSD post hoc tests, when indicated. For post-stimulation analysis of blood pressure and heart rate, the data were subtracted from the pre-stimulation value; then, a linear mixed effects model was conducted using group, time after injection, and the interaction term as fixed effects and animal identifiers as a random effect. If indicated, least squared means for group, time, or the interaction term were compared using multiple comparison that was corrected for compounding type I errors using Tukey’s HSD adjustment (R Studio, lsmeans package).

All data are presented as mean ± 95% confidence interval (CI) from the corresponding regression models (ANOVA or linear mixed effects model).

## Results

### Physiological parameters

**Fig. 1:**
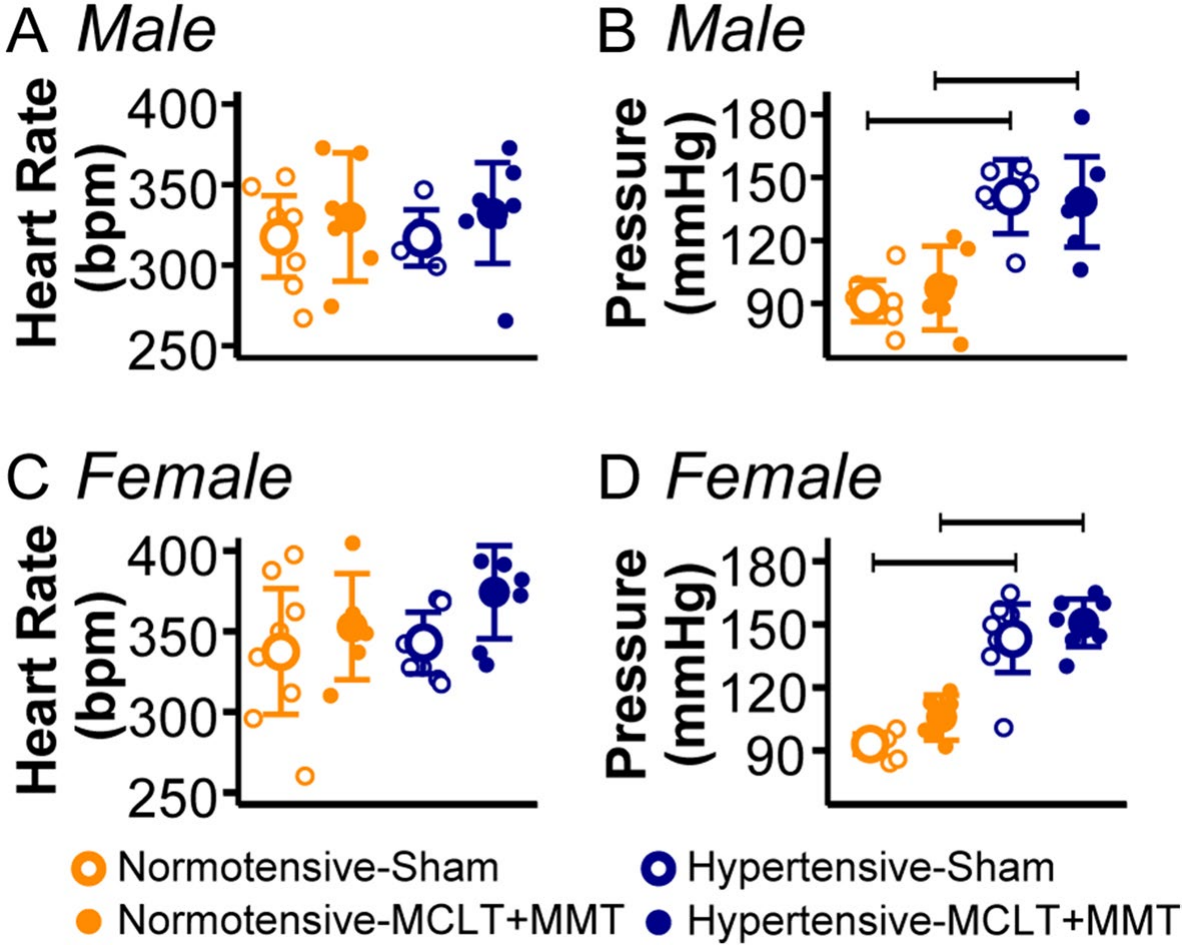
Resting cardiovascular measures taken at the endpoint in males (top) and females (bottom), including heart rate (**A**, **C**) and blood pressure (**B**, **D**). Blood pressure measures were statistically different for all hypertensive animals, with no differences due to the surgical group. Bars indicate *p* < 0.05 between the groups. Data are presented as mean ± 95% confidence interval

Animal weights over the course of the study are presented in Supplemental Fig. 2. In males, weights increased over time (week main effect, *p* < 0.001) at different rates (week-group interaction, *p* < 0.001); however, these differences were due to strain differences, with no effect of surgery. Similarly, females weighed more over time (week main effect, *p* < 0.001). In addition to strain differences in females, normotensive-sham animals weighed more than normotensive-MCLT+MMT females (*p* = 0.03); however, this may have been driven by differences in baseline. At the endpoint, both male and female hypertensive animals had increased blood pressure compared to normotensive rats, as expected (Fig. 1B, D; *p*’s < 0.001); however, no differences were seen in heart rate between normotensive and hypertensive animals (Fig. 1A, C). Moreover, MCLT+MMT surgery did not affect blood pressure or heart rate in either the normotensive or hypertensive animals (*p*’s > 0.05).

### Tactile allodynia

Supplemental Fig. 3 describes the tactile sensitivity following sham and MCLT+MMT surgery in both male normotensive and hypertensive rats (top) and in female normotensive and hypertensive rats (bottom). For males, paw withdrawal thresholds in the hypertensive-sham group (23.34 ± 3.86 grams) were lower than the normotensive-sham group (30.46 ± 3.72 grams; *p* = 0.01). Similarly, male hypertensive-MCLT+MMT (23.57 ± 3.94 grams) had lower paw withdrawal thresholds than male normotensive-MCLT+MMT animals (29.45 ± 3.96 grams, *p* = 0.048). For females, no differences were seen in paw withdrawal thresholds (group main effect, *p* = 0.077).

### Activity

Supplemental Fig. 4 shows the activity patterns of animals with sham and MCLT+MMT surgery in both male normotensive and hypertensive rats (top) and in female normotensive and hypertensive rats (bottom). For males, hypertensive animals (76 ± 5%, hypertensive-sham; 74 ± 4%, hypertensive-MCLT+MMT) spent less time in corners compared to normotensive controls (83 ± 4%, normotensive-sham; 90 ± 5%, normotensive-MCLT+MMT), regardless of the surgical type (sham *p* = 0.036, MCLT+MMT *p* < 0.001). Normotensive-MCLT+MMT (90 ± 5%) rats also tended to spend more time in corners compared to normotensive-sham (83 ± 4%) animals; however, this difference was not statistically significant (*p* = 0.057). In male animals, it appears that hypertensive animals traveled further distances; however, this was not statistically significant (group main effect *p* = 0.051).

For females, hypertensive animals (67 ± 4%, hypertensive-sham; 66 ± 4%, hypertensive-MCLT+MMT) spent less time in corners compared to normotensive controls, regardless of surgery group (sham *p* < 0.001, MCLT+MMT *p* < 0.001). In addition, normotensive-MCLT+MMT animals (84 ± 4%) spent more time in corners compared to normotensive-sham controls (78 ± 4%, *p* = 0.045). For females, normotensive-MCLT+MMT animals traveled less distance than both normotensive-sham and hypertensive-MCLT+MMT animals (normotensive-MCLT+MMT vs. normotensive-sham, *p* = 0.023; normotensive-MCLT+MMT vs. hypertensive-MCLT+MMT, *p* = 0.045).

### Corticosterone

Corticosterone measures are reported in Supplemental Fig. 5. For both male and female animals, no differences were observed in serum corticosterone measurements (*p*’s > 0.05).

### Histology

**Fig. 2:**
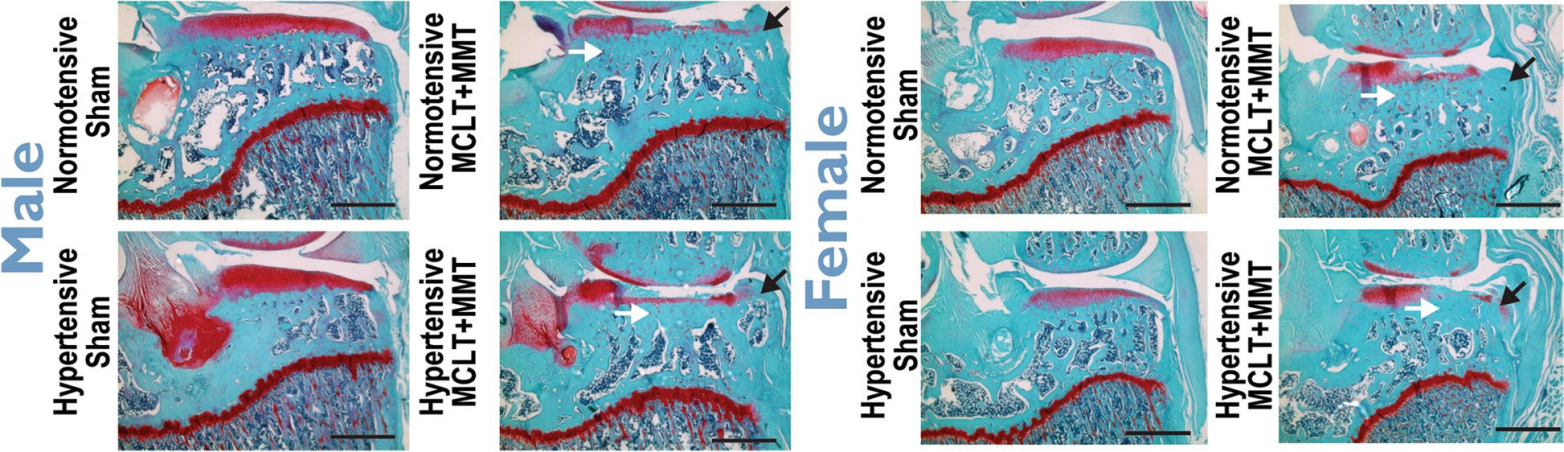
Representative images of joint histology. Black arrows indicate osteophyte formation, and white arrows point out regions of subchondral bone remodeling. Scale bar = 1 mm

**Fig. 3:**
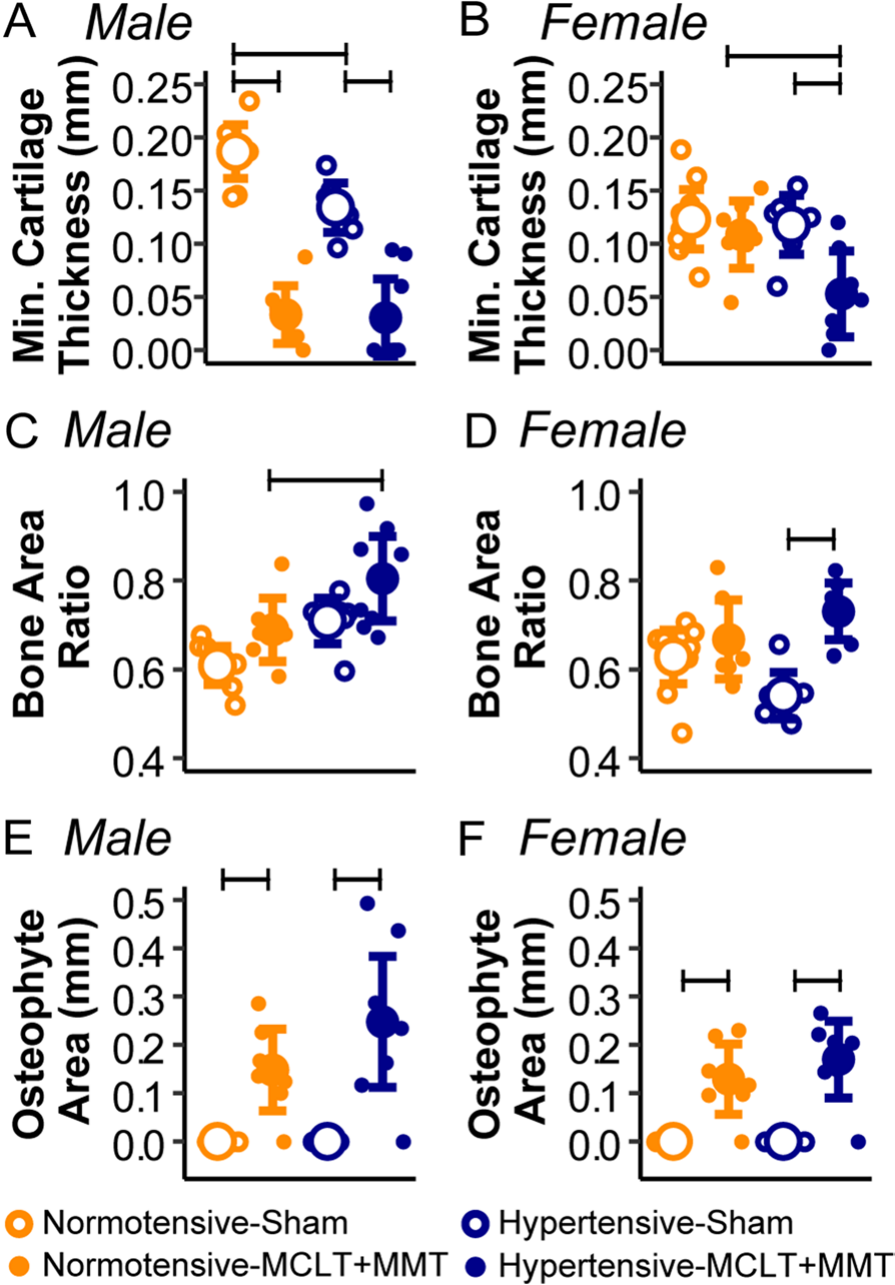
Quantitative histological results for male (left) and female (right) tibial region in the medial joint compartment. **A** In males, minimum cartilage thickness decreased with MCLT+MMT compared to sham in both hypertensive and normotensive animals. **B** In females, decreased cartilage thickness due to MCLT+MMT only occurred in hypertensive animals. **C** The ratio of the bone area associated with the subchondral bone plate to the total area of bone and trabecular bone in the subchondral space (“bone area ratio”) increased in hypertensive-MCLT+MMT males compared to normotensive-MCLT+MMT males. **D** Hypertensive-MCLT+MMT females also had an increased bone area ratio; however, this was compared to hypertensive-sham animals. **E**, **F** Osteophyte areas increased due to MCLT+MMT in both strains and sexes. Bars represent *p* < 0.05 between the groups. Data are presented as mean ± 95% confidence interval

Representative histological images are presented in Fig. 2, with quantification presented in Fig. 2 and scoring in Fig. 3. In males, MCLT+MMT surgery resulted in smaller minimum cartilage thickness in both hypertensive (0.13 ± 0.04 mm, hypertensive-sham; 0.03 ± 0.024 mm, hypertensive-MCLT+MMT) and normotensive animals (0.19 ± 0.03mm, normotensive-sham; 0.03 ± 0.03 mm, normotensive-MCLT+MMT, Fig. 3A; sham *p* = 0.024, hypertensive *p* < 0.001, normotensive *p* < 0.001). However, in females, decreases in minimum cartilage thickness were only present in hypertensive animals. Specifically, hypertensive-MCLT+MMT (0.05 ± 0.028 mm) was lower than both hypertensive-sham (0.12 ± 0.04 mm) and normotensive-MCLT+MMT animals (0.11 ± 0.04 mm, Fig. 3B; *p* = 0.013, *p* = 0.038, respectively).

The ratio of the subchondral bone area associated with the subchondral bone plate and trabecular bone is reported in Fig. 3C, D. In male animals, this bone area ratio did not differ between normotensive-sham and normotensive-MCLT+MMT, nor did this ratio differ between hypertensive-sham and hypertensive-MCLT+MMT animals. However, male hypertensive-MCLT+MMT rats (0.80 ± 0.06) exhibited increased bone area ratios compared to normotensive-MCLT+MMT rats (0.69 ± 0.08, *p* = 0.043), indicating a greater portion of the subchondral bone area is dedicated to the calcified trabecular bone rather than marrow. The magnitude of this shift in bone area ratio was similar between hypertensive-sham (0.71 ± 0.08) and hypertensive-MCLT+MMT (0.80 ± 0.06) animals, but these changes were not statistically different (*p* = 0.095). In females, hypertensive-MCLT+MMT (0.73 ± 0.06) animals had larger bone area ratios than hypertensive-sham animals (0.54 ± 0.08, *p* < 0.001).

In both males and females, MCLT+MMT surgery resulted in the formation of osteophytes, though no statistical differences were found between the size of these osteophytes in hypertensive and normotensive rats (Fig. 3E, F).

**Fig. 4:**
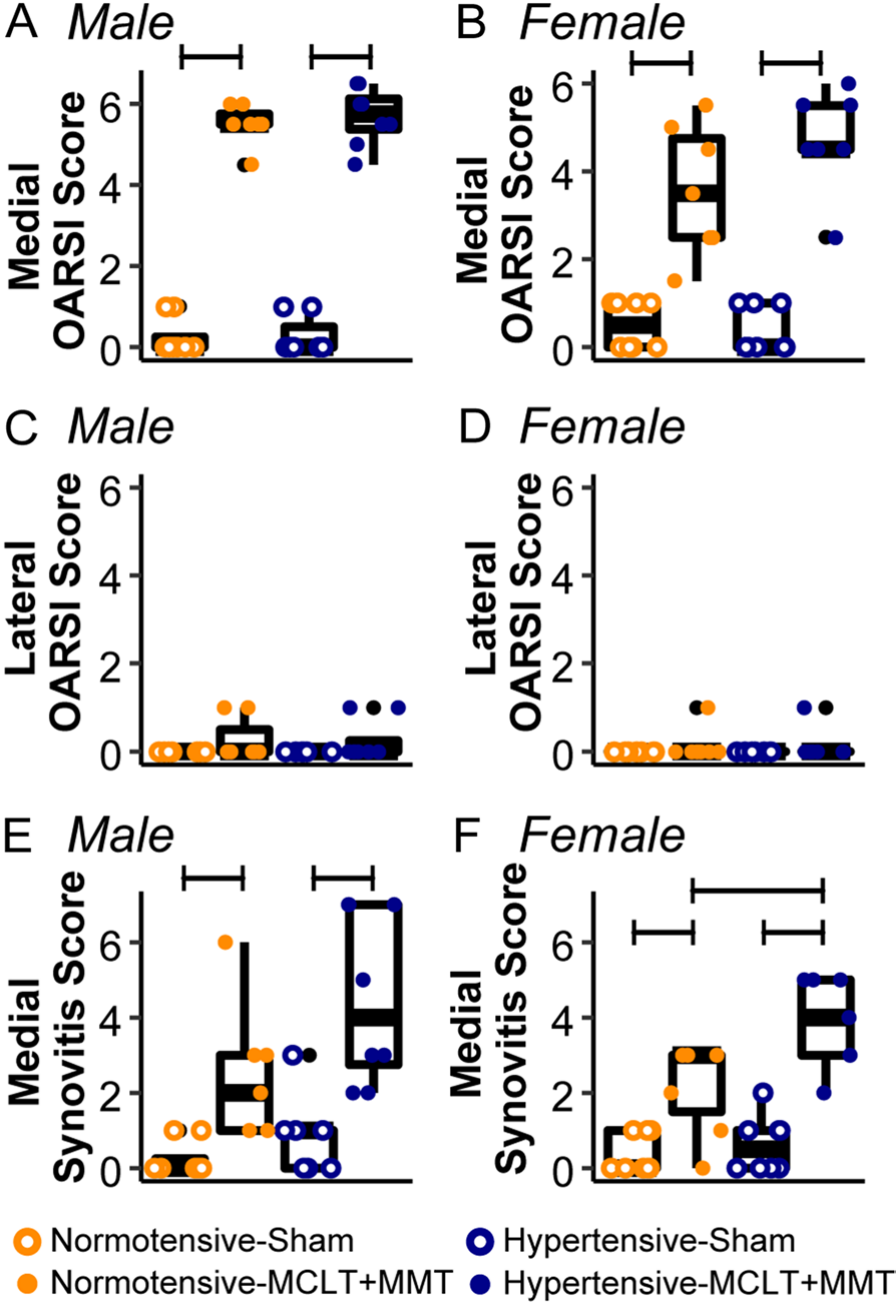
Results of semi-quantitative histological scoring for males (left) and females (right). All OARSI scores were determined based on the tibial region of the joint. **A** In males, medial compartment OARSI scores increased in both normotensive-MCLT+MMT and hypertensive-MCLT+MMT animals compared to their sham controls. **B** In females, this increase in medial compartment OARSI scores for the MCLT+MMT groups was also observed. **C**, **D** Lateral compartment OARSI scores did not change in any group. **E** In males, low-grade synovitis was present in both MCLT+MMT groups. **F** In females, low-grade synovitis was present in both MCLT+MMT groups, with hypertensive-MCLT+MMT animals having higher synovitis scores than normotensive-MCLT+MMT animals. Bars represent *p* < 0.05 the between groups. Black dots represent the outliers of the boxplots, while colored dots represent the individual data points. Data are presented as median and interquartile range

In addition to quantitative scoring of manually defined regions of interest, histological sections were semi-quantitatively scored. Male MCLT+MMT animals displayed increased OARSI scores relative to sham controls, with no differences due to hypertension (Fig. 4A; normotensive-MCLT+MMT vs. normotensive-sham, *p* = 0.002; hypertensive-MCLT+MMT vs. hypertensive-sham, *p* = 0.002). Similarly, female MCLT+MMT animals displayed increased OARSI scores relative to sham controls, with no differences due to hypertension (Fig. 4B; normotensive-MCLT+MMT vs. normotensive-sham, *p* = 0.002; hypertensive-MCLT+MMT vs. hypertensive-sham, *p* = 0.002). Interestingly, female normotensive-MCLT+MMT animals had a lower median (3.5, IQR = 2.25) than hypertensive-MCLT+MMT animals (3.5, IQR = 1), but this was not statistically significant (*p* = 0.21). No differences in OARSI score were seen for the lateral compartment, for any group of either sex (Fig. 4C, D). No differences were seen between animals that received meloxicam and those that did not (*p*’s > 0.05).

Synovitis was scored in the medial compartment of affected limbs using the Krenn Scale. In males, low-grade synovitis was present in normotensive-MCLT+MMT animals (2, IQR = 2) compared to normotensive-sham controls (0, IQR = 0.25; *p* = 0.008; Fig. 4E). Similarly, low-grade synovitis was present in hypertensive-MCLT+MMT animals (4, IQR = 4.25) compared to hypertensive-sham controls (2, IQR = 2; *p* = 0.008; Fig. 4E). The difference between normotensive-MCLT+MMT and hypertensive-MCLT+MMT was not statistically significant (*p* = 0.08). In females, low-grade synovitis was present in normotensive-MCLT+MMT animals (3, IQR = 1.5) compared to normotensive-sham controls (0, IQR = 1; *p* = 0.025; Fig. 4F). Similarly, low-grade synovitis was present in hypertensive-MCLT+MMT animals (4, IQR = 2) compared to hypertensive-sham controls (0.5, IQR = 1; *p* = 0.044; Fig. 4F). Additionally, hypertensive-MCLT+MMT females exhibited higher synovitis scores compared to normotensive-MCLT+MMT females (*p* = 0.046). No differences were seen between animals that received meloxicam and those that did not (*p*’s > 0.05).

### Endpoint measures of cardiovascular autonomic function

**Fig. 5:**
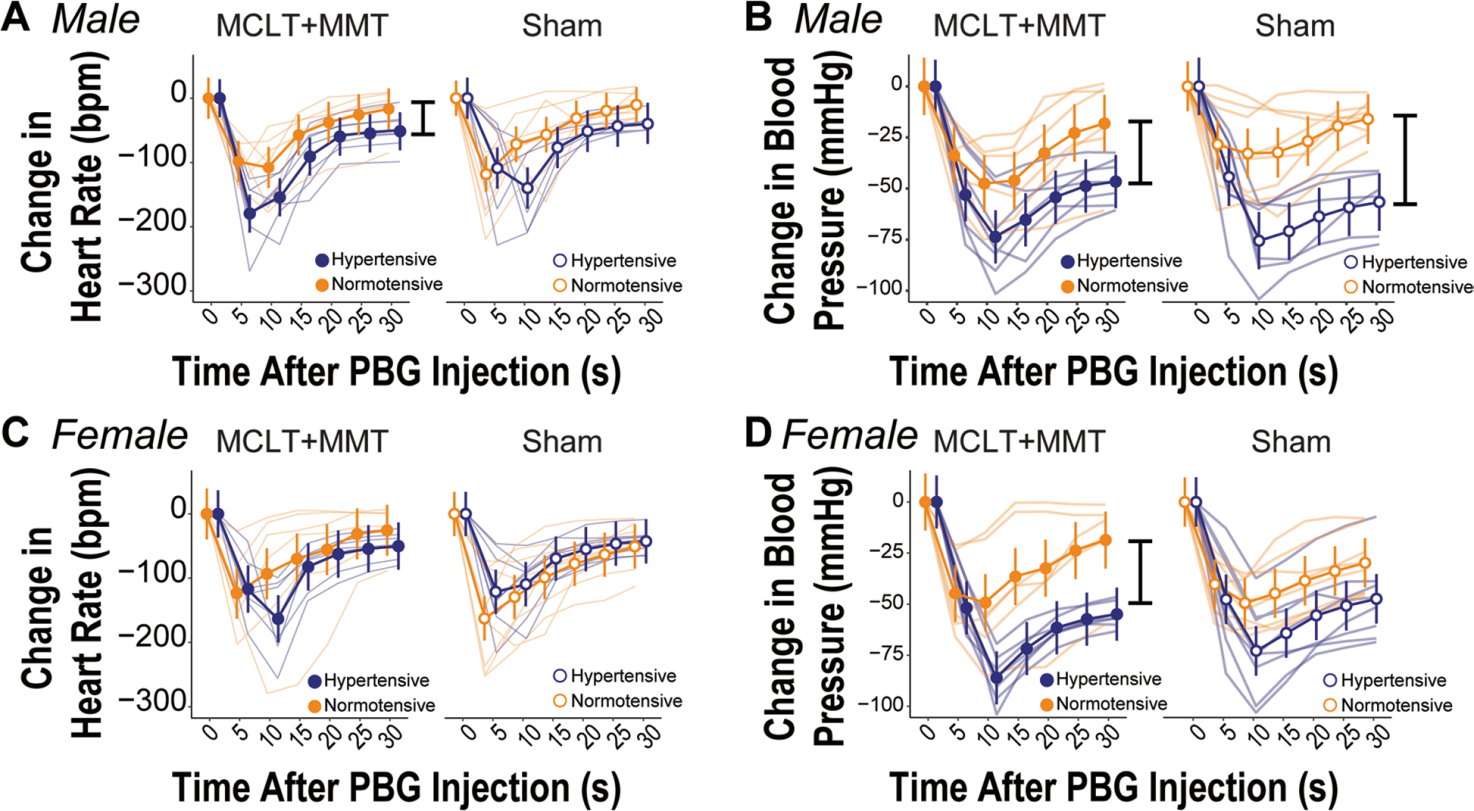
Cardiovascular responses to chemical stimulation of vagal afferents with 1-phenylbiguanide (PBG) in males (top) and females (bottom). **A** In males, heart rate responses were increased in the hypertensive-MCLT+MMT group compared to normotensive-MCLT+MMT; this decrease was not statistically significant in the male sham groups. **B** In males, blood pressure responses to PBG were enhanced with hypertension, regardless of the surgical group. **C** No differences were seen in female heart responses to PBG; however, **D** hypertension resulted in larger blood pressure drops with PBG administration in the MCLT+MMT surgical groups. This difference was not statistically significant in the sham surgical group. Bars indicate *p* < 0.05 between the groups. Data are presented as mean ± 95% confidence interval

Male hypertensive animals had increased drops in blood pressure due to intravenous phenybiguanide administration in both MCLT+MMT (− 48.8 ± 11.0 mmHg, hypertensive-MCLT+MMT; − 28.7 ± 11.9 mmHg, normotensive-MCLT+MMT; *p* = 0.018) and sham surgery (− 52.9 ± 11.9 mmHg, hypertensive-sham; − 22.3 ± 10.3 mmHg, normotensive-sham; *p* < 0.001, Fig. 5B). There were no differences between the surgical groups within the same rat strain (*p*’s > 0.05). In females (Fig. 5D), hypertensive-MCLT+MMT animals (− 54.8 ± 11.5 mmHg) had larger drops in blood pressure compared to normotensive-MCLT+MMT animals (− 29.3 ± 12.4 mmHg, *p* = 0.005). Similar magnitude shifts occurred in hypertensive-sham animals (− 48.3 ± 10.8 mmHg) compared to normotensive-sham animals (− 33.8 ± 10.8 mmHg); however, this difference was not statistically significant (*p* = 0.06).

Male hypertensive-MCLT+MMT animals (− 84.2 ± 20.9 bpm) had a larger decrease in heart rate due intravenous administration of phenybiguanide compared to normotensive-MCLT+MMT animals (− 49.0 ± 22.6 bpm, *p* = 0.03). These heart rate shifts were not significant in the male sham surgical groups (Fig. 5A). Importantly, this shift in heart rate in male hypertensive-MCLT+MMT animals appeared to be driven by the heart rate response at 5 s after phenybiguanide injection. In females, there were no apparent differences in heart rate responses to phenybiguanide injection due to surgery or strain (Fig. 5C). Supplemental Fig. 6 is provided as an alternative visualization to Fig. 5, to more easily compare differences to both surgery and strain.

## Discussion

In this study, we explored the interactions between comorbid hypertension and OA development in both male and female rats. Endpoint measures of cardiovascular function confirmed that all hypertensive animals had increased blood pressure, with no differences due to the surgical group. Additionally, comorbid hypertension and OA appear to alter joint remodeling differently in male and female rats, where female rats exhibited an increased depth of cartilage loss and greater levels of synovitis and male rats exhibited more subchondral bone remodeling. Hypertensive animals also exhibited enhanced blood pressure responses to pharmacological activation of the vagus nerve. Taken together, these data suggest an influence of hypertension on the development of OA joint pathology. While pain and anxiety-related behavioral results were inconclusive in this study, these data motivate future mechanistic studies on the influence of hypertension on OA pathophysiology and symptoms.

The development of symptomatic and radiographic knee OA is associated with hypertension in humans [24]. In our study, male hypertensive rats with OA had higher trabecular bone area ratios compared to male normotensive rats with OA, indicating either a greater extent of bone marrow resorption or the development of subchondral bone sclerosis. This is similar to a study by Chan et al., where naïve 40-week-old male SHR animals developed greater bone volume fractions, as shown by μCT analysis, compared to a normotensive control [25]. Furthermore, bone area ratios in female normotensive rats displayed no difference due to surgical induction of OA; however, in female hypertensive rats with OA, bone area ratios increased compared to the sham control. Combined, these data indicate that comorbid hypertension and OA lead to more severe subchondral bone pathology following a meniscal injury. Furthermore, this remodeling could be related to vascular etiology in the subchondral bone, which has previously been proposed as a mechanism for OA development [26]. As such, these questions and the mechanisms driving these changes may be further dissected with an in-depth analysis of subchondral bone pathology in hypertensive and normotensive rats. For example, three-dimensional analysis through μCT would also provide greater insight into the differences in subchondral bone pathology throughout the whole joint, not only the loading region.

While surgical induction of OA in male animals resulted in significant cartilage damage (indicated by smaller minimum cartilage thickness), no differences in cartilage damage were observed between hypertensive and normotensive animals in the OA group. This finding is in contrast to a prior study in hypertensive mice with exercise-induced OA, where strenuous exercise combined with hypertension resulted in larger Mankin scores compared to non-exercise and normotensive controls [27]. The discrepancy in results may be due to the model of OA. Here, surgical induction of OA led to full-depth cartilage lesions at 8 weeks. Examining an earlier stage of disease, before full-depth lesions appear, may reveal exacerbated cartilage pathology in male animals. Furthermore, in this prior work, larger Mankin scores positively correlated with the number of angiotensin II receptor type I positive cells in the joint, suggesting a role for the renin-angiotensin system in the development of joint pathology in male animals. While we did not observe increased cartilage damage in male hypertensive animals, female hypertensive animals with OA did have reduced cartilage thickness compared to both hypertensive animals with a sham surgery and normotensive animals with OA. This finding in female hypertensive animals somewhat parallels the findings in male hypertensive mice by Yamagishi et al. [27] Moreover, this loss of cartilage thickness in hypertensive females may reveal a potential explanation of the sex differences in OA development in rodents, where female animals tend to develop joint damage at a slower rate than males if at all [28, 29]. Combined, this may support a sex-dependent role for autonomic dysfunction in the susceptibility of the joint to post-traumatic OA.

Systemic administration of phenylbiguanide was used as a pharmacological activator of the vagus nerve, thereby causing a drop in the heart rate and blood pressure for all animals. In males, pre-existing hypertension led to exaggerated drops in blood pressure in both OA and non-OA animals. However, for females, these exaggerated drops in blood pressure were only observed in animals with pre-existing hypertension and OA. Again, these findings may help explain potential sex-dependent effects on OA development and progression that arise from systemic physiologic differences.

Anti-hypertensive drugs have been associated with decreased odds of developing knee OA in humans [30], and thus, targeting autonomic function may provide some potential to modify the progression of OA. Here, systolic pressure and pulse pressure, but not diastolic pressure, have been associated with the development of knee OA [30], and persons taking three or more anti-hypertensive medications have decreased odds of knee OA compared to persons taking no drugs [30]. Our work in this study, along with work by others in hypertensive rodents, provide evidence of shared pathophysiology between OA and hypertension. These overlaps provide the groundwork for future studies evaluating if and how OA progression may be controlled or slowed via anti-hypertensive drugs.

In contrast to our current findings, we previously conducted a similar study using male Lewis rats [15] where heart rate responses to intravenous phenybiguanide administration did not significantly differ between OA and non-OA. However, the Lewis rat has a dysfunctional hypothalamic-pituitary-adrenal axis (HPA axis), which can confound autonomic measures [31]. In contrast, outbred Sprague-Dawley rats have enhanced HPA axis function relative to Lewis [32] and have been used as controls in studies of HPA axis hyperactivity [33]. This prior work was a motivation for this study using both hypertensive and normotensive animals. Moreover, unlike literature in mice that has shown slowed progression of OA in females [28, 29], Lewis rats do not develop meaningful sex differences in joint pathology [34]. Combined, our work continues to question the general use of the Lewis rat strain to model knee OA pathogenesis, particularly when not also evaluating the systemic effects of a dysfunctional HPA axis.

In association with HPA axis functions, serum cortisol levels increase with exposure to stress. In some chronic pain states, which may be considered a type of chronic stress, basal serum cortisol is sustained at higher levels [35]. As such, these elevated systemic cortisol levels have been related to the presence of ongoing pain [36]. However, the association between chronic OA pain and cortisol remains unclear [37], particularly in preclinical models. In this study, bi-weekly corticosterone measurements did not reveal meaningful differences with either chronic hypertension or the surgical induction of knee OA. Previously, SHR animals were found to have higher basal corticosterone levels compared to normotensive Wistar rats in samples taken immediately after euthanasia without anesthesia [38]. However, we did not observe similar findings between SHR rats and normotensive Sprague-Dawley rats. A possible confounder of our data was the use of isoflurane anesthesia during serum collection, as isoflurane has been reported to affect corticosterone levels in female animals [39]. Because cortisol is increased with acute stress, future studies may consider evaluating the responsiveness of the system through the measurement of serum corticosterone levels before and after a stressful event. Moreover, hair-based corticosterone tests may provide better data on the long-term corticosterone levels in rats and reduce the variability caused by diurnal fluctuations in cortisol in the serum.

In this work, we attempted to measure the effects of pre-existing hypertension on knee OA symptoms, and the MCLT+MMT model has previously produced detectable decreases in paw withdrawal thresholds across different strains, sexes, and laboratories [34, 40–42]. We did not observe evidence of tactile allodynia in this study. Some confounding factors may have contributed to this finding. First, the estrus phase of female animals affects withdrawal thresholds [43], and this study did not control for this factor due to logistical constraints. Additionally, male scents can result in a higher stress state in females and thereby affect female responses [44]. Again, due to logistical constraints, male and female animals were not tested separately in this study, and all von Frey testing was conducted by a male experimenter. Moreover, SHR animals have shown modality-dependent hyperalgesia or hypoalgesia that is likely related to autonomic control of blood pressure and not sustained high blood pressure per se [45]. Though the current data of tactile allodynia in comorbid hypertension and OA are inconclusive, future refined studies are warranted to explore the autonomic influence on pain-related behaviors. These studies should more carefully control for confounding experimental variables and should consider other pain assessment modalities, such as thermal pain or non-evoked assays.

In knee OA studies, open-field activity has been more commonly used as a non-evoked pain assay to assess exploratory activity [46]. Here, reductions in distance traveled is as a surrogate measure of movement-evoked pain (or avoidance of movement-evoked pain). In this study, appreciable differences between the groups were limited to the female normotensive OA group, where normotensive OA animals moved less compared to the other groups, possibly indicating higher levels of movement-evoked pain. Anxiety-related behaviors can also be assessed in open-field environments, where time spent in different parts of the arena is used as surrogate measures of anxiety. This interpretation of open-field results is less common in studies of OA, despite the common comorbid presentation of anxiety and depression in patients with OA and chronic musculoskeletal pain. Moreover, SHR animals are known to typically display less anxiety-like behaviors and higher levels of locomotion [47, 48]. In our data, there were no differences between SHR and normotensive animals at baseline, but at week 2, normotensive animals with MCLT+MMT surgery begin to spend more time in corners, potentially associated with higher levels of anxiety.

Our work in this study, along with work by others in hypertensive rodents, provides evidence of shared pathophysiology between OA and hypertension. Demonstration of altered OA pathogenesis in the SHR animal provides opportunities for mechanistic studies that relate OA pathogenesis to hypertension in the future. For example, as mentioned above, vascular remodeling in the subchondral bone has been suggested as a contributor to OA development. Additionally, future studies may examine the renin-angiotensin system in more depth. Finally, as autonomic nervous system dysregulation has been hypothesized to be a factor in the development of both OA and hypertension, more mechanistic analysis of autonomic function in comorbid OA and hypertension is warranted.

Finally, a potential limitation of this study is the use of two different rat strains to test our hypothesis. First, SHR animals are an inbred line that was originally developed from the outbred Wistar Kyoto but has since genetically diverged [49, 50]. Thus, while Wistar Kyoto rats are commonly the control for SHR studies, significant concerns have been raised about the applicability of this control [49, 50]. As there are only a handful of papers that study OA development in Wistar Kyoto rats, we selected Sprague-Dawley rats as the normotensive control for this study, though genetic differences between the two rat strains remain a potential confounder and limitation of our study. Additionally, large weight differences are noted between SHR and Sprague-Dawley rats, which may contribute to OA pathology, However, it would be hypothesized that higher weights (i.e., Sprague-Dawley) would lead to worsened pathology, yet the results of the present study suggest that the lower weights (i.e., SHR) had worsened pathology. Moreover, it is unknown how changes in activity level in SHR animals affect OA joint pathology. The magnitude of the activity shift in SHR animals in this study is unlikely to extrapolate to overuse of the joint, though this is a possible confounder in the experiment. For these reasons, future studies should consider an induction of hypertension, such as the deoxycorticosterone acetate (DOCA)—salt model [51], within the same rat strain rather than rely on genetic models of hypertension.

## Conclusions

In conclusion, hypertension may contribute to exacerbated OA pathophysiology in a sex-dependent manner. Furthermore, hypertension, but not OA, resulted in exaggerated drops in blood pressure with pharmacological activation of the vagus nerve, indicating dysfunctional autonomic responses with hypertension. Combined, these data suggest possible autonomic influences on the development and progression of comorbid hypertension and OA. Future studies should further clarify the effects on OA joint pathology due to hypertension. Furthermore, additional work is needed to identify possible shared mechanisms underlying hypertension and OA development. Additionally, due to the inconclusive nature of the behavioral results in this study, the role of hypertension on OA pain, disability, and activity should be further assessed using refined behavioral techniques.

## Supplementary Information

**Additional file 1: Supplemental Figure 1.** Visual representation of study design. SHR = spontaneously hypertensive rat; MCLT+MMT = medial collateral ligament transection + medial meniscus transection.

**Additional file 2: Supplemental Figure 2.** Animal weights over the course of the study. (A) Male rats displayed increases in weight over time, with normotensive Sprague Dawley rats weighing more than hypertensive SHR. No differences were noted due to MCLT+MMT surgery. (B) Similarly, females weighed more over time with normotensive Sprague Dawley rats weighing more than hypertensive SHR. Additionally, normotensive-MCLT+MMT females weighed less than normotensive-Sham females; however, this difference was present at baseline as well. Dashed lines indicate *p*<0.05 between both hypertensive groups vs. both normotensive groups. Solid lines indicate *P*<0.05 between specific groups. Data are presented as mean ± 95% confidence interval.

**Additional file 3: Supplemental Figure 3.** Ipsilateral paw withdrawal threshold, as assessed via von Frey, in (A) male and (B) female animals. Male hypertensive-sham animals had lower paw withdrawal thresholds than male normotensive-sham animals and male hypertensive-MCLT+MMT animals had lower thresholds than normotensive-MCLT+MMT animals. No other appreciable differences were noted between groups. Data are presented as mean ± 95% confidence interval.

**Additional file 4: Supplemental Figure 4.** Percent time spent in corners of open-field arena, a measure of anxiety-related behavior, in (A) male and (C) female animals and total distance travelled in (B) male and (D) female rats. Regardless of sex, hypertensive-MCLT+MMT animals spent less time in corners. In females, this decrease extended to the sham group. In female normotensive animals, MCLT+MMT resulted in more time spent in corners. In male animals, it appears that hypertensive animals travelled further distances; however, this was not statistically significant (group main effect *p*=0.051). For females, normotensive-MCLT+MMT animals travelled less distance than both normotensive-sham and hypertensive-MCLT+MMT animals. Data are presented as mean ± 95% confidence interval.

**Additional file 5: Supplemental Figure 5.** Longitudinal blood serum corticosterone levels in (A) male and (B) female animals, with no meaningful differences due to hypertension nor surgery. Data are presented as mean ± 95% confidence interval.

**Additional file 6: Supplemental Figure 6.** Cardiovascular responses to chemical stimulation of vagal afferents with 1-phenylbiguanide (PBG) in males (top) and females (bottom). (A) In males, heart rate responses were increased in the hypertensive-MCLT+MMT group compared to normotensive-MCLT+MMT; this decrease was not statistically significant in the male sham groups. (B) In males, blood pressure responses to PBG were enhanced with hypertension, regardless of surgical group. (C) No differences were seen in female heart responses to PBG; however, (D) hypertension resulted in larger blood pressure drops with PBG administration in the MCLT+MMT surgical groups. This difference was not statistically significant in the sham surgical group. Bars indicate *p*<0.05 between groups. Data are presented as mean ± 95% confidence interval.

## Data Availability

The data that support the findings of this study are available from the corresponding author, KDA, upon reasonable request.

## Abbreviations

OA: Osteoarthritis
ANS: Autonomic nervous system
HPA: Hypothalamic pituitary adrenal
SHR: Spontaneously hypertensive rat
MCLT: Medial collateral ligament transection
MMT: Medial meniscus transection
UF: University of Florida
