# Supplementary figures and images for: Hypertension contributes to exacerbated osteoarthritis pathophysiology in rats in a sex-dependent manner
Source: Arthritis Res Ther. 2023 Jan 12;25:7. doi: 10.1186/s13075-022-02966-9 (PMC9835335; doi:10.1186/s13075-022-02966-9)

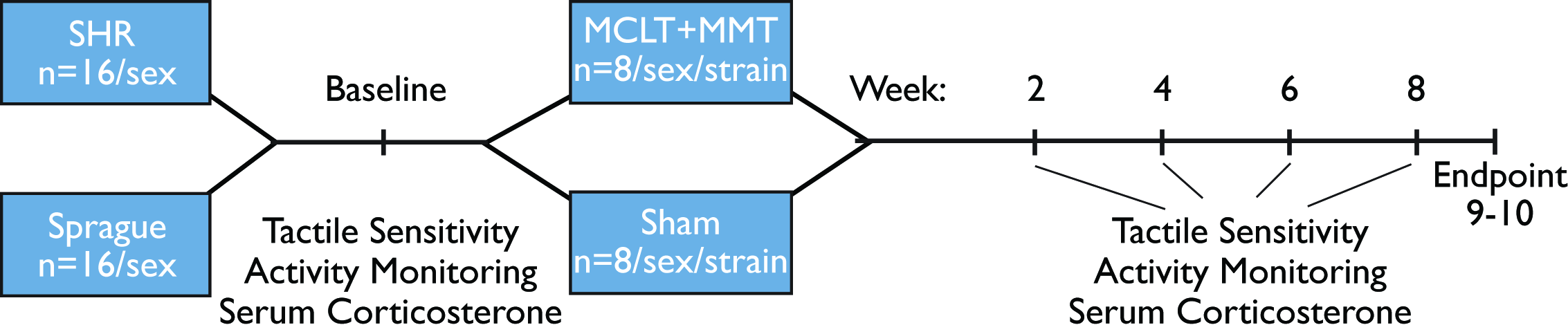

Supplement: Supplementary file 1 — Additional file 1: Supplemental Figure 1. Visual representation of study design. SHR = spontaneously hypertensive rat; MCLT+MMT = medial collateral ligament transection + medial meniscus transection. [file 13075_2022_2966_MOESM1_ESM.tif]

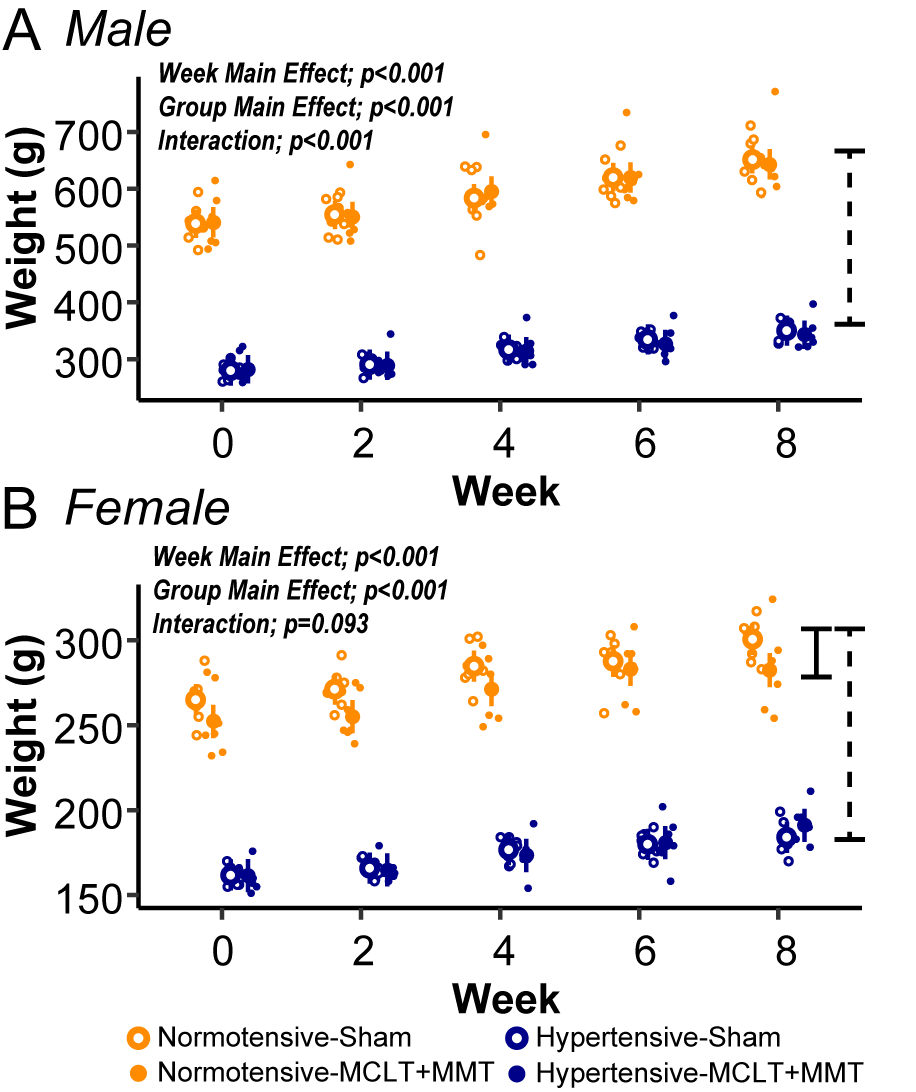

Supplement: Supplementary file 2 — Additional file 2: Supplemental Figure 2. Animal weights over the course of the study. (A) Male rats displayed increases in weight over time, with normotensive Sprague Dawley rats weighing more than hypertensive SHR. No differences were noted due to MCLT+MMT surgery. (B) Similarly, females weighed more over time with normotensive Sprague Dawley rats weighing more than hypertensive SHR. Additionally, normotensive-MCLT+MMT females weighed less than normotensive-Sham females; however, this difference was present at baseline as well. Dashed lines indicate p<0.05 between both hypertensive groups vs. both normotensive groups. Solid lines indicate P<0.05 between specific groups. Data are presented as mean ± 95% confidence interval. [file 13075_2022_2966_MOESM2_ESM.tif]

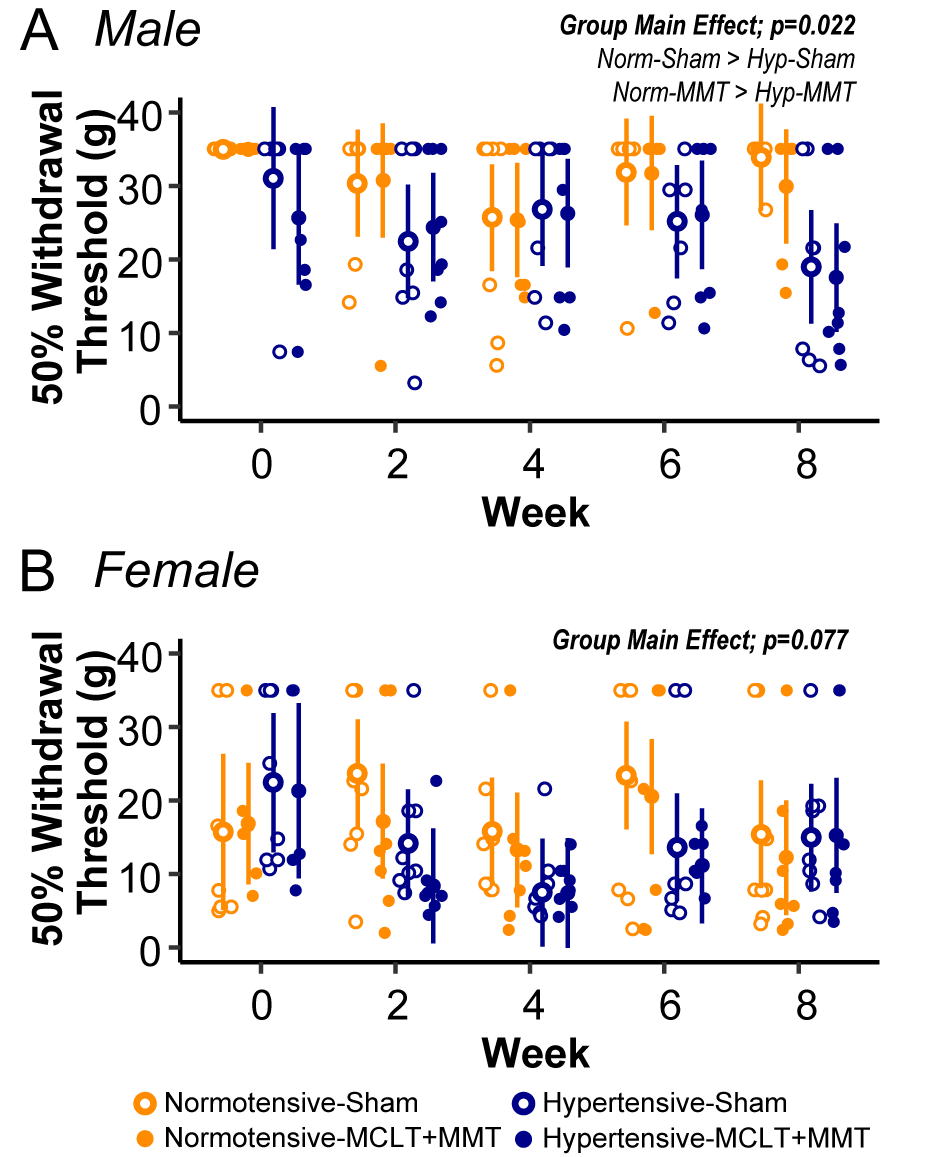

Supplement: Supplementary file 3 — Additional file 3: Supplemental Figure 3. Ipsilateral paw withdrawal threshold, as assessed via von Frey, in (A) male and (B) female animals. Male hypertensive-sham animals had lower paw withdrawal thresholds than male normotensive-sham animals and male hypertensive-MCLT+MMT animals had lower thresholds than normotensive-MCLT+MMT animals. No other appreciable differences were noted between groups. Data are presented as mean ± 95% confidence interval. [file 13075_2022_2966_MOESM3_ESM.tif]

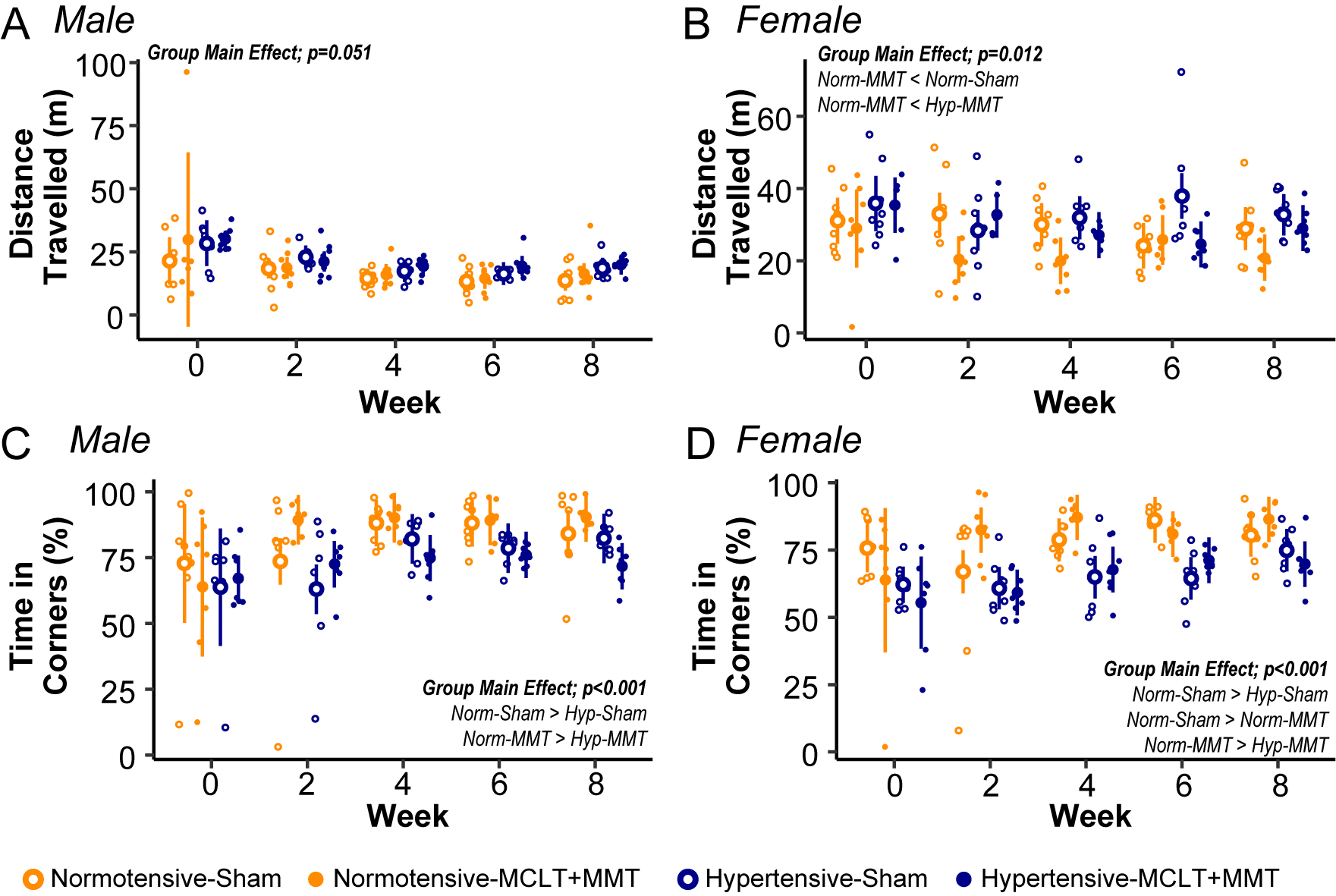

Supplement: Supplementary file 4 — Additional file 4: Supplemental Figure 4. Percent time spent in corners of open-field arena, a measure of anxiety-related behavior, in (A) male and (C) female animals and total distance travelled in (B) male and (D) female rats. Regardless of sex, hypertensive-MCLT+MMT animals spent less time in corners. In females, this decrease extended to the sham group. In female normotensive animals, MCLT+MMT resulted in more time spent in corners. In male animals, it appears that hypertensive animals travelled further distances; however, this was not statistically significant (group main effect p=0.051). For females, normotensive-MCLT+MMT animals travelled less distance than both normotensive-sham and hypertensive-MCLT+MMT animals. Data are presented as mean ± 95% confidence interval. [file 13075_2022_2966_MOESM4_ESM.tif]

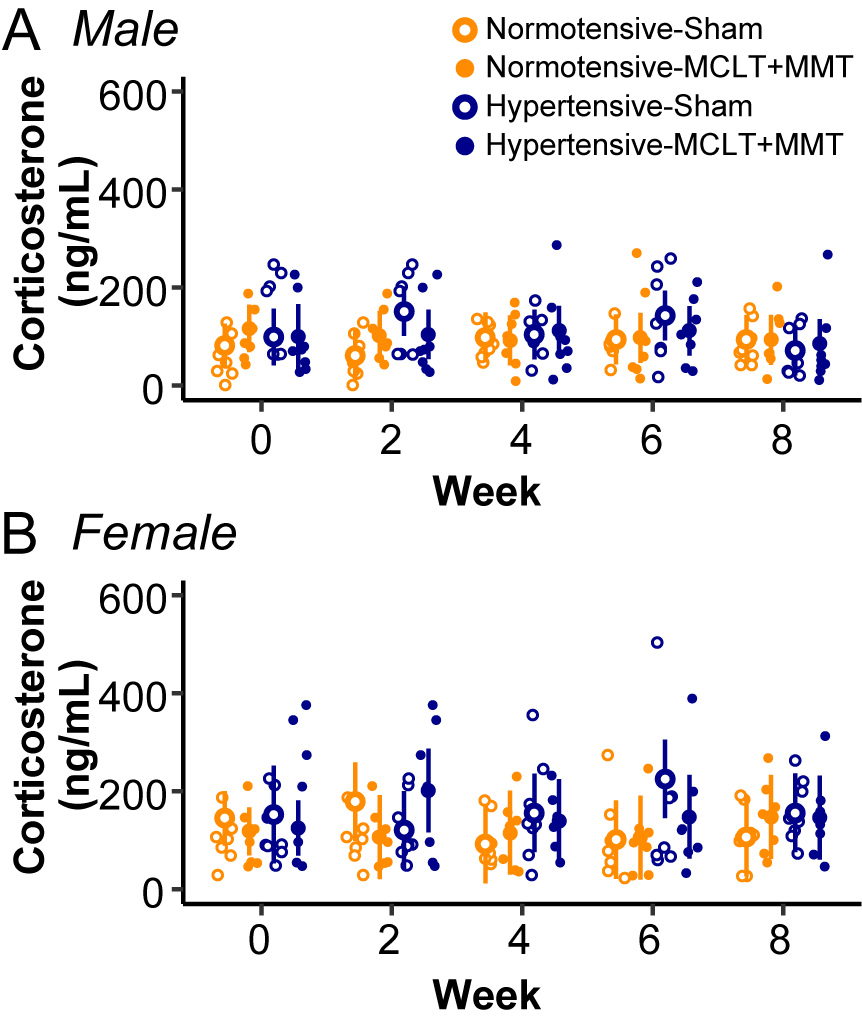

Supplement: Supplementary file 5 — Additional file 5: Supplemental Figure 5. Longitudinal blood serum corticosterone levels in (A) male and (B) female animals, with no meaningful differences due to hypertension nor surgery. Data are presented as mean ± 95% confidence interval. [file 13075_2022_2966_MOESM5_ESM.tif]

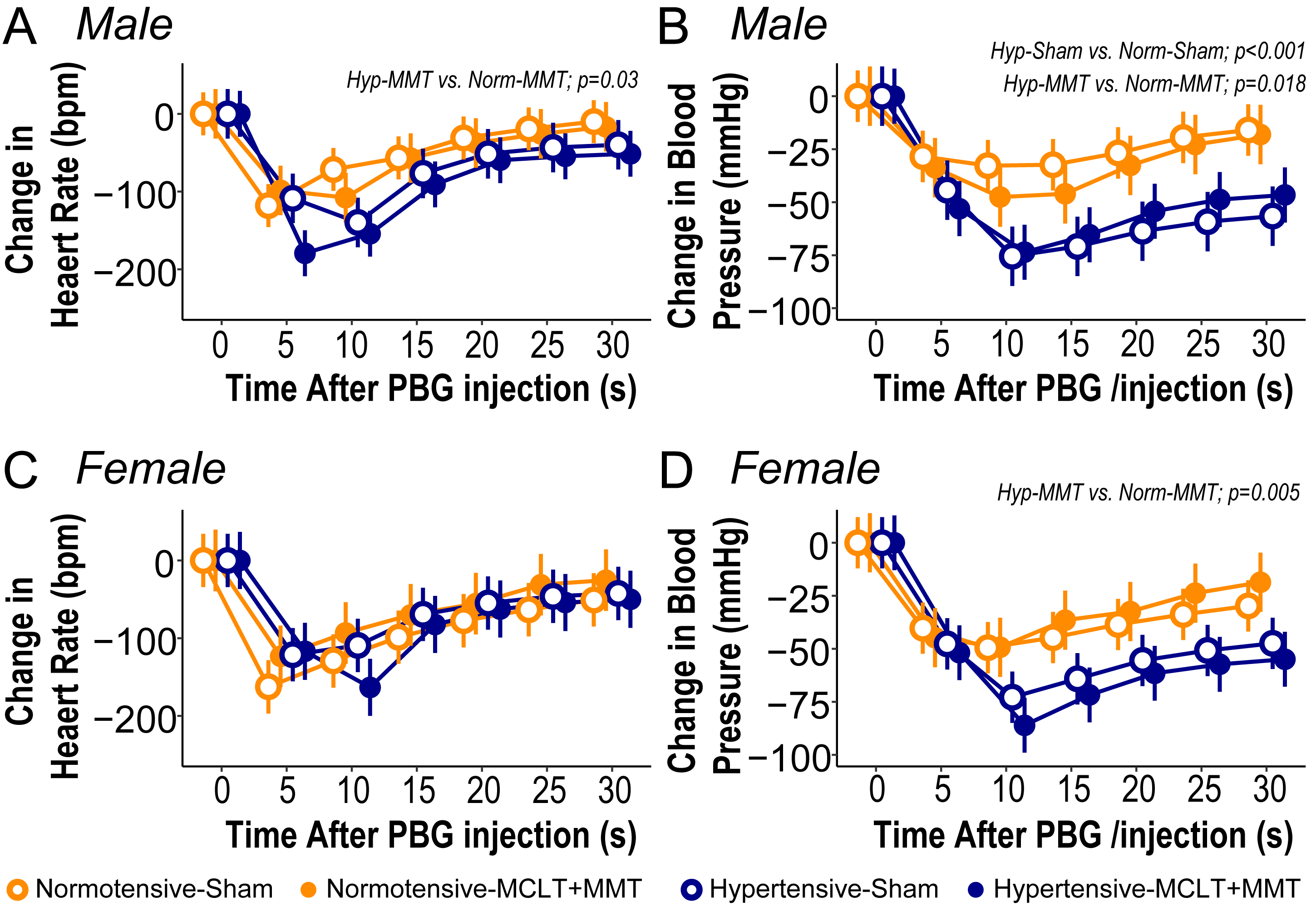

Supplement: Supplementary file 6 — Additional file 6: Supplemental Figure 6. Cardiovascular responses to chemical stimulation of vagal afferents with 1-phenylbiguanide (PBG) in males (top) and females (bottom). (A) In males, heart rate responses were increased in the hypertensive-MCLT+MMT group compared to normotensive-MCLT+MMT; this decrease was not statistically significant in the male sham groups. (B) In males, blood pressure responses to PBG were enhanced with hypertension, regardless of surgical group. (C) No differences were seen in female heart responses to PBG; however, (D) hypertension resulted in larger blood pressure drops with PBG administration in the MCLT+MMT surgical groups. This difference was not statistically significant in the sham surgical group. Bars indicate p<0.05 between groups. Data are presented as mean ± 95% confidence interval. [file 13075_2022_2966_MOESM6_ESM.tif]
